# Graphene-driving novel strain relaxation towards AlN film and DUV photoelectronic devices

**DOI:** 10.1038/s41377-022-00861-1

**Published:** 2022-05-30

**Authors:** Hieu. P. T. Nguyen

**Affiliations:** New Jersey Institute of Technology, Department of Electrical & Computer Engineering, Newark, NJ 07102 USA

**Keywords:** Inorganic LEDs, Optical properties and devices

## Abstract

Graphene-driving strain-pre-store engineering enables the epitaxy of strain-free AlN film with low dislocation density for DUV-LED and the unique mechanism of strain-relaxation in QvdW epitaxy was demystified.

## Abstract (web summary)

Graphene-driving strain-pre-store engineering enables the epitaxy of strain-free AlN film with low dislocation density for high efficiency DUV-LEDs.

## Graphical abstract

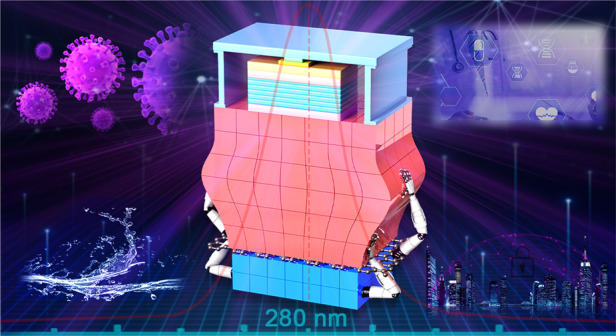

Group III-nitride semiconductor owns direct energy bands and wide bandgap and thus can be widely used in high-efficiency ultraviolet (UV) photo-electronic emitters and detectors^1–3^. Recently, UV light-emitting diode (UV-LED) based on AlGaN has received tremendous attention due to its broad applications including sterilization, polymer curing, biochemical detection, non-line-of-view communication, and special lighting^4,5^. Compared with traditional UV light-source using mercury, xenon, argon, deuterium, or excimer, UV-LEDs provide several advantages such as mercury-free environmental protection, compact and portable, low power consumption, low operating voltage, etc.^6^. In 1998, Han et al.^7^ utilized Al_0.2_Ga_0.8_N/GaN as a multiple quantum well (MQW) structure and successfully demonstrated the world’s first UV-LED with a wavelength shorter than 360 nm. However, its light output power (LOP) is only 13 μW at a current of 20 mA, and the external quantum efficiency (EQE) is less than 1%. In 2006, Taniyasu et al.^8^ reported that AlN based *p*-type/intrinsic/*n*-type (PIN) and metal-insulator-semiconductor (MIS) LEDs showed a peak luminous wavelength at 210 nm, which was the shortest luminous wavelength ever obtained using III-nitride semiconductor, with an EQE of only 6–10%. Hirayama et al.^9^ reported the record EQE exceed 20% at 20 mA for an emission wavelength of 275 nm. Over the past two decades, AlGaN-based deep UV LEDs (DUV-LEDs) have made significant progress both in terms of LOP and EQE. From the current overall situation, the EQE reported for DUV-LEDs is mostly below 10% or even 5%, which still has a lot of room for improvement compared with the full-fledged longer wavelength near-ultraviolet and blue LEDs.

Among the multiple factors that restrict the qualitative leap in the photo-electronic properties of DUV-LEDs, the epitaxial quality of the structure is the most prominent part. The AlN film, which is the basic template layer of AlGaN-based DUV-LEDs, is usually epitaxially grown on a hetero-substrate due to its lack of cost-effective homogenous substrate^10^. Due to the intractable problem of mismatch between the substrate and the AlN epilayer, heteroepitaxy will inevitably introduce various defects inside the epilayers^11,12^. In this regard, several techniques have been proposed and great progress have been made in the epitaxial growth of AlN templates, such as epitaxial lateral overgrowth (ELO) technology on patterned sapphire substrates (PSS) and patterned AlN/sapphire templates including micron-sized and nano-sized patterns^13,14^. The above method does reduce the dislocation density of the AlN epilayer to a certain extent, but it is helpless to eradicate the residual stress inside the crystal, which will lead to the nonuniformity of the Al distribution in the upper AlGaN layer accompanied by wafer bending, severely limiting the device performance^15,16^.

A recent publication by Chang et al.^17^ reported the successful realization of strain-free AlN films with low dislocation density by graphene-driven strain pre-storage engineering and demonstrated its application on high-performance DUV-LED. More importantly, the research team also proposed a unique strain relaxation mechanism in quasi-van der Waals (QvdW) epitaxy, different from the previous “interface displacement” theory previously thought^18^, which was expected to allow continuous modulation of the strain state of the QvdW epitaxy AlN film by adjusting the initial nucleation morphology of the epilayers. The key strategy for growing high-quality strain-free AlN films on the graphene layer is presented in Figure 2a of this paper^17^. Briefly, N_2_ plasma treatment was used to convert graphene grown on sapphire to defect-rich graphene, so that the nucleation of AlN mainly occurred at N-deficient sites through Al–N bond as the fulcrum. Subsequently, AlN was densely nucleated on defect-rich graphene, and then rapidly expanded into films by graphene-driven vdW epitaxy. By characterizing a series of crystal qualities and stress states of the as-grown AlN films with various thicknesses, the researchers found that the dislocation density of epilayer illustrated an anomalous sawtooth-like evolution, instead of a simple decreasing with the increase of film thickness (Fig. 3b)^17^. More importantly, the presence of graphene provided an additional source of tensile strain for the epitaxy system compared to its counterpart on bare sapphire, resulting in the final graphene-based strain-free AlN film, as shown in Fig. 3f^17^. Meanwhile, the DFT calculation results demonstrated that the graphene-driven nucleation of AlN with small-size high-density during the initial growth process pre-stored additional tensile strain at the initial stage of growth, which just offseted the compressive strain effect of the hetero-mismatch. Further, the as-fabricated AlN/graphene-based DUV-LED exhibited excellent performance in terms of structural quality, luminous efficiency, and wavelength stability, proving that this growth strategy can effectively improve the photo-electronic properties of the device.

The photo-electronic performance of DUV-LED devices is generally limited by critical issues associated with the poor quality and large strain of the nitride material system caused by the inherent mismatch of heteroepitaxy. This work achieves a strain-free AlN film with low dislocation density by graphene-driving strain-pre-store engineering and demonstrates its application on high-performance DUV-LED. Through both experimental and theoretical analysis, the researchers reveal the unique mechanism of strain-relaxation in QvdW epitaxy, and state that it is expected to realize further modulation of epilayer with various strain states by adjusting the nucleation density of initial AlN growth. The valuable perceptions of this work reveal multiple beneficial effects of graphene on nitride growth, which not only provides useful inspiration for the diversified development of 2D materials, but also provides reliable way for developing the practical application of graphene in the fields of nitride-based optoelectronic and high-power electronic devices.
